# Increased Sensitivity of DNA Damage Response-Deficient Cells to Stimulated Microgravity-Induced DNA Lesions

**DOI:** 10.1371/journal.pone.0125236

**Published:** 2015-04-27

**Authors:** Nan Li, Lili An, Haiying Hang

**Affiliations:** 1 Key Laboratory for Protein and Peptide Pharmaceuticals, Institute of Biophysics, Chinese Academy of Sciences, Beijing, China; 2 University of Chinese Academy of Sciences, Beijing, China; Cornell University, UNITED STATES

## Abstract

Microgravity is a major stress factor that astronauts have to face in space. In the past, the effects of microgravity on genomic DNA damage were studied, and it seems that the effect on genomic DNA depends on cell types and the length of exposure time to microgravity or simulated microgravity (SMG). In this study we used mouse embryonic stem (MES) and mouse embryonic fibroblast (MEF) cells to assess the effects of SMG on DNA lesions. To acquire the insight into potential mechanisms by which cells resist and/or adapt to SMG, we also included *Rad9*-deleted MES and *Mdc1*-deleted MEF cells in addition to wild type cells in this study. We observed significant SMG-induced DNA double strand breaks (DSBs) in *Rad9* ^-/-^ MES and *Mdc1* ^-/-^ MEF cells but not in their corresponding wild type cells. A similar pattern of DNA single strand break or modifications was also observed in *Rad9* ^-/-^ MES. As the exposure to SMG was prolonged, *Rad9* ^-/-^ MES cells adapted to the SMG disturbance by reducing the induced DNA lesions. The induced DNA lesions in *Rad9* ^-/-^ MES were due to SMG-induced reactive oxygen species (ROS). Interestingly, *Mdc1* ^-/-^ MEF cells were only partially adapted to the SMG disturbance. That is, the induced DNA lesions were reduced over time, but did not return to the control level while ROS returned to a control level. In addition, ROS was only partially responsible for the induced DNA lesions in *Mdc1* ^-/-^ MEF cells. Taken together, these data suggest that SMG is a weak genomic DNA stress and can aggravate genomic instability in cells with DNA damage response (DDR) defects.

## Introduction

The effects of space environment on human health are a major concern for manned space exploration. The integrity of genomic DNA is important for normal physiological functions of cells and DNA damage is related to many diseases such as cancer and aging [1, 2]. A statistically significant increase in the yield of chromosomal aberrations in lymphocytes from cosmonauts at their first long-term space missions has been reported [3]. Ohnishi et al. observed the accumulation of p53 (an important DNA damage sensing molecule) in the skin and the muscle of rats, and increased DNA damage in human cancer cells cultured during space flight [4]. Microgravity and space radiation are two important factors in space environment. It is well known that ionizing irradiation (IR) can lead to DNA damage. The effect of space radiation, especially the radiation of high-energy particles of high atomic mass (HZE) which are much more prevalent in space than on the earth, has aroused much attention. However, little is known about the effect of microgravity on cellular DNA damage.

Because of the heavy costs and the limited access to space flight, simulated microgravity (SMG) on earth has been widely used in space life research. Previously, we found that SMG alone was unable to induce increased DNA damage in wild type mouse embryonic stem (MES) cells after 2 days of exposure [5]. Degan et al. reported that exposure of freshly drawn lymphocytes and lymphoblastoid cells to SMG for 24 or 72 hours is not significantly associated with the induction of DNA damage [6]. However, Kumari et al. reported that the exposure of human lymphocytes to SMG for 7 days significantly increased the level of DNA damage [7]. Roberts et al. also observed that human retinal pigment epithelial cells exposed to SMG for 24 hours suffered from significant damage in the form of single-stranded DNA breaks when compared with control cells [8]. It seems that the effect of SMG on DNA damage varies among different types of cells and depends on the time length of microgravity exposure. To counteract DNA damage, eukaryotes have evolved the DNA damage response (DDR). The DDR is carried out by a set of complex pathways that sense DNA damage and transduce the information to the cells to coordinate cellular responses to DNA damage [9]. We hypothesize that SMG is a weak stress on cellular DNA. The final effects of SMG on DNA damage are determined by both the length of SMG exposure time and DDR status of the cell. The efficiency of the DDR system varies depending on the cell type and genetic background. This might be the reason why the researchers obtained the contradictory results mentioned above. Thus, studies using cell models with a deficient DDR system which are sensitive to DNA damaging agents might provide us with clearer information.

Rad9 and Mdc1 are two important components of the DDR network [10–14]. We used Rad9 deficient mouse embryonic stem cells and Mdc1 deficient mouse embryonic fibroblast (MEF) cells to investigate the effects of SMG on DNA damage in this study. This design was based on the following considerations. Ataxia telangiectasia mutated (ATM) and ATM-and RAD3 related (ATR) pathways are the cores of DDR network [15]. Rad9 plays important roles in ATR-dependent Chk1 activation [16] while Mdc1 acts as a signal amplifier of the ATM pathway [17]. It is not known which pathway participates in the response to SMG so both pathways were included in this study. Moreover, Rad9 also directly participates in base excision repair (BER), nucleotide excision repair (NER), mismatch repair (MMR) and homologous recombination repair (HR) [18–28]. *Rad9* ^-/-^ MES cells demonstrated a remarkable increase in spontaneous chromosome aberrations and hypoxanthine-guanine phosphoribosyltransferase (*HPRT*) mutations, and were extremely sensitive to DNA damage agents such as UV light, gamma rays and hydroxyurea relative to wild type *Rad9* ^*+/+*^ MES controls[11]. *Mdc1*-deficient mice have been shown to be hypersensitive to IR [29]. Therefore, the *Rad9* ^-/-^ MES and *Mdc1* ^-/-^ MEF cells with DDR defects are sensitive models for assessing the effects of SMG on DNA damage.

In this study, we systematically investigated the effects of SMG on mammalian cell DNA damage using *Rad9* ^-/-^ MES cells, *Mdc1* ^-/-^ MEF cells and their corresponding wild type cells. We observed significant SMG-induced DNA damage in *Rad9* ^-/-^ MES and *Mdc1* ^-/-^ MEF cells but not in wild type cells. We found that SMG-induced reactive oxygen species (ROS) was at least partially responsible for DNA damage in these cells.

## Materials and Methods

### 3D-clinostat

The 3D-clinostat, a multidirectional G force generator used for SMG treatment, was produced by Center for Space Science and Applied Research, Chinese Academy of Sciences[30]. By employing a simultaneous rotation on two axes, the 3D-clinostat is able to produce an environment with an average of 10^–3^ G, thus simulating microgravity conditions.

### Cell culture

*Rad9* ^*+/+*^ MES cells, *Rad9* ^*-/-*^ MES cells and *Rad9* ^*-/-*^ MES cells with ectopic expression of mRad9 (*Rad9* ^*-/-*^+mRad9 MES cells) were obtained from Lieberman’s laboratory [11, 31]. *Rad9* ^*+/+*^ and *Rad9* ^*-/-*^ MES cells were maintained on gelatin-coated flasks in standard MES cell medium in the presence of leukemia inhibitory factor (LIF) without a feeder layer [32]. *Rad9* ^*-/-*^ MES cells were transfected with pZeoSV2-mRad9 and then challenged with 100 mg/ml zeocin to generate stable mutant cells ectopically expressing mRad9. The selected *Rad9* ^*-/-*^+mRad9 MES cells were cultured in the medium containing 25 mg/ml zeocin to maintain the transfected genes in the cells [33]. *Mdc1* ^*+/+*^ and *Mdc1* ^*-/-*^ MEF cells were obtained from Chen’s laboratory [17]. The cells were cultured in DMEM (Invitrogen) supplemented with 10% fetal bovine serum (Hyclone) and 100U/ml penicillin/streptomycin (Gibco).

The cells were seeded in culture flasks (Becton Dickinson) and cultured under a 1G (the gravity on the earth) environment for 18 hours to achieve adhesion. Then the flasks were filled with fresh and 5% CO_2_-balanced complete medium to eliminate air bubbles and to diminish turbulence as well as shear forces. The flasks were sealed air-tight. The samples were randomized to two groups. One group was cultured in the 3D-clinostat (group SMG) and the other was cultured in 1G environment (group 1G). The system was maintained at 37°C. The day on which the cells were mounted on the clinostat was referred to as Day 0. The culture medium was not changed during the experimental period.

### Apoptosis assays

MES cells were seeded at a concentration of 5×10^5^ cells per 25 cm^2^ culture flask. Cultured cells were trypsinized for 3 min using 0.1% trypsin at 37°C (Sigma), washed twice with cold PBS, and resuspended in 1× binding buffer [10 mmol/l HEPES (pH 7.4), 140 mmol/l NaCl, and 2.5 mmol/l CaCl_2_] at a concentration of 1×10^6^ cells per milliliter. Then cells were stained with Alexa Fluor 488 annexin V and PI (Invitrogen) for 15 min at room temperature, before flow cytometric analysis.

### Comet assay

The protocol published by Singh et al. [34] was used with minor modifications. The slides were pre-coated with a thin layer of 1% normal melting agarose and allowed to dry. Single cell suspensions of either SMG-treated or control cells were harvested and resuspended to 5×10^5^ cells/ml. Twenty μl of each final suspension was added to 80 μl of pre-melted 0.75% low melting agarose and was pipetted onto the pre-coated slide. After solidification, the slides were placed in neutral or alkaline lysis solution and the cells were lysed in the dark at 4°C for 2 hours. Slides were then placed in 1×TBE (for neutral comet assay)/alkaline (for alkaline comet assay) buffer in the dark at 4°C for 30 min to allow for unwinding of the DNA. The slides were subjected to electrophoresis at ~0.74 V/cm for 30 min. Following electrophoresis, the slides were stained with propidium iodine (PI). Fluorescence images were captured using a microscope and analyzed by CASP-1.2.2 software (University of Wroclaw) for tail moment (the geometric mean of fluorescence on the tail from the Nucleus).

### ROS activity assays

Intracellular ROS activity was analyzed by staining the cells with 10 mM 2^',^7^'^2dichlorodihydrofluorescin diacetate (DCF-DA) (Sigma, USA) [35]. The assay employed the cell-permeable fluorogenic probe DCF-DA, which diffused into cells and was deacetylcated by cellular esterases into the non-fluorescent DCFH. In the presence of ROS, DCFH was rapidly oxidized to highly fluorescent DCF. The fluorescence intensity was measured by flow cytometry (FACSCalibur, Becton Dickinson, USA) with excitation and emission settings of 488 and 530 nm, respectively. For antioxidant treatment, *Rad9* ^-/-^ MES and *Mdc1* ^-/-^ MEF cells were mock-treated or treated with 0.05, 0.1 or 0.5 mM N-acetylcysteine under SMG for 1 day.

### Flow cytometric assessment of Histone H2AX Phosphorylation

Flow cytometric assessment of γ-H2AX formation was performed according to Huang et al.[36] with minor modifications. *Rad9* ^*+/+*^ and *Rad9* ^*-/-*^ MES cells were fixed in 1 ml of 75% ethanol at -20°C for at least 2 hours and resuspended in 2 ml of PBS plus 1% BSA (w/v) and 0.2% Triton X-100 (BSA-T-PBS) at room temperature for 5 min. Then the cells were incubated with anti-γ-H2AX antibody (Upstate) at 4°C overnight, rinsed with cold BSA-T-PBS twice and stained with fluorescent-conjugated secondary antibodies (Molecular Probes) at room temperature for 1 hour. Flow cytometric analyzes were performed on a FACSCalibur (Becton Dickinson).

### Quantitative real-time PCR analysis

Total RNA was isolated with RNeasy Mini Kit (Qiagen) following the manufacturer's protocol. For reverse transcription-PCR (RT-PCR), 2 μg total RNA were reverse transcribed in a reaction volume of 20 μl to form cDNA using the SuperScript First-Strand Synthesis System (Invitrogen). Real-time PCR was performed using the StepOnePlus system (ABI) with SYBR Green I (Takara) to label amplified DNA. A standard curve method of quantification was used to calculate the expression of target genes relative to the housekeeping gene GAPDH. Experiments were performed three times. The following primer pairs were used for the PCR reactions: Nox2, 5’-TGTGGTTGGGGCTGAATGTC-3’ and 5’-CTGAGAAAGGAGAGC AGATTTCG-3’; Nox4, 5’-GAAGGGGTTAA ACACCTCTGC-3’ and 5’-ATGCTCTGCTTAAACACAATCCT-3’ GAPDH, 5’- AGGTCGGTGTGAACGGATTTG-3’ and 5’-TGTAGACCATGTA GTTGAGGTCA-3’. PCR procedures for these genes were template denaturation at 94°C for 1 min, then 40 cycles of 94°C for 15 sec, 57°C for 20 sec, 72.0°C for 20 sec, and a final extension at 72°C for 3 min.

### Western blotting analysis

The cell lysate was prepared in 1× SDS-sample buffer, from a final concentration of 10^4^ cells per microliter. Ten micrograms of protein were resolved on a 10% SDS-PAGE gel, and proteins were transferred to a polyvinylidene difluoride membrane. The membrane was probed consecutively with primary and peroxidase-conjugated secondary antibodies, and the signal was detected using the SuperSignal West Pico Chemiluminescence Substrate system(Pierce), Primary and Secondary antibodies used in this study were mouse anti-GAPDH (Kang Chen), rabbit anti-NOX2 (Abcam), peroxidase-conjugated anti-rabbit IgG (sigma) and anti-mouse IgG (sigma). The mean normalized optical density (OD) of Nox2 protein band relative to the OD of GAPDH band from the same sample was calculated using ImageJ software.

### Antioxidant enzyme activity assays

The activity of superoxide dismutase (SOD), glutathione peroxidase (GSH-Px), and catalase (CAT) in MES cell lysates were determined with assay kits (BioAssay Systems) according to the manufacturer’s protocols. The antioxidant enzyme activity of each sample was defined as the change in optical density and normalized to that of the sample of *Rad9* ^+/+^ MES cells cultured under 1G for 1day.

### Cell number expansion analysis

Mouse embryonic stem cells were seeded at a concentration of 3×10^4^ cells per 25 cm^2^ culture flask and the samples were handled as described in “Cell culture”. Mouse embryonic fibroblast cells were seeded at a concentration of 10^5^ cells per 25 cm^2^ culture flask and the samples were handled as mentioned in “Cell culture”. At the indicated time points (day 1, day 2, day 3, day 4 and day 5) the attached cells were collected by trypsinization and the cell numbers were determined using a hemocytometer.

### Statistical analysis

Data were expressed as mean ± SD. The Student’s t-test was performed to determine statistical significance of the differences. A P value of < 0.05 was considered statistically significant.

## Results

### Requirement of Rad9 and Mdc1 to prevent DNA damage in DDR deficient cells experiencing SMG

**Fig 1:**
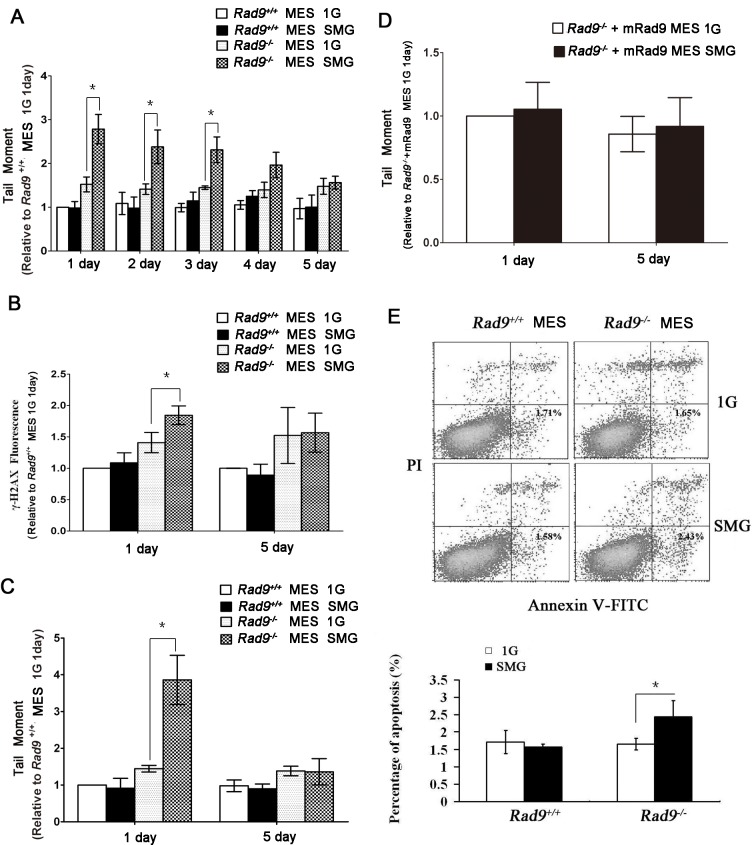
Effects of SMG on DNA damage and apoptosis in *Rad9* ^*+/+*^ and *Rad9* ^*-/-*^ MES cells. (A) Evaluation of DNA double strand break by neutral comet assay in *Rad9* ^***+/+***^ and *Rad9* ^***-/-***^ MES cells cultured under 1G or SMG condition. Time points were 1, 2, 3, 4 and 5 days. At least 50 cells for each condition were scored for comet tail moment. (B) Flow cytometric analysis of γ-H2AX formation in *Rad9* ^***+/+***^ and *Rad9* ^***-/-***^ mMES cells cultured under 1G or SMG condition for 1 or 5 days. (C) Evaluation of DNA damage by alkaline comet assay in *Rad9* ^***+/+***^ and *Rad9* ^***-/-***^ MES cells cultured under 1G or SMG condition for 1 or 5 days. At least 50 cells for each condition were scored for comet tail moment. (D) Evaluation of DNA damage by alkaline comet assay in *Rad9* ^***-/-***^ MES cells with ectopic expression of *Rad9* (*Rad9* ^***-/-***^ *+Rad9* MES cells) cultured under 1G or SMG condition for 1 or 5 days. At least 50 cells for each condition were scored for comet tail moment. (E) Flow cytometric analysis of *Rad9* ^***+/+***^ and *Rad9* ^***-/-***^ MES cells cultured under 1G or SMG condition for 1 day to assess apoptosis using Annexin V labeling. Experiments were performed three times and representative analyses are shown (upper). The lower part is the quantitative comparison of apoptosis between the 1G Group and the SMG Group. The data represent mean ± SD of at least three independent experiments. Student’s t test, *P<0.05.

Previously, we reported that SMG alone could not induce DNA damage in wild type MES cells after 2 days of exposure [5]. *Rad9* ^-/-^ MES cells were shown to be extremely sensitive to DNA damaging agents compared with *Rad9* ^*+/+*^ MES cells [11]. Here, *Rad9* ^-/-^ MES cells were used as a sensitive model to assess the effect of SMG on DNA damage. *Rad9* ^*+/+*^ MES cells and *Rad9* ^*-/-*^ MES cells were cultured under 1G and SMG for 1, 2, 3, 4 and 5 days, respectively, and the levels of DNA double strand breaks (DSBs) in these cultured cells were assessed by neutral comet assay. As shown in Fig 1A, there was no statistically significant increase in DSB levels in wild type MES cells under either 1G or SMG. Interestingly, DSB levels in *Rad9* ^*-/-*^ MES cells were significantly higher under SMG than those under 1G for 1, 2 and 3 days. There was a trend that the enhancement of the SMG-induced DNA lesions in *Rad9* ^*-/-*^ MES cells was gradually attenuated with increasing culture time under SMG, and the increased DSB levels after 4 or 5 days under SMG were no longer statistically different from those under 1G, suggesting that an adaptive response is gradually established through time under SMG and reduces DSBs.

Phosphorylated histone H2AX (γ-H2AX) is found at the site of nascent DNA DSBs, and the γ-H2AX antibody is frequently used to monitor DSBs [36]. As shown in Fig 1B, the DSB levels in *Rad9* ^*-/-*^ MES cells cultured under SMG for 1 day were statistically significantly higher than those in cells maintained under 1G for 1 day, and this difference disappeared after 5 days of culture under SMG. *Rad9* ^*+/+*^ MES cells did not demonstrate a significant difference of DSB levels between the 1G group and the SMG group for either 1 day or 5 days. Altogether, these results indicate that SMG induces increased DSBs in *Rad9* ^*-/-*^ MES cells at the early stage of treatment.

The alkaline comet assay can be used to measure DNA lesions including single and double strand breaks and base modifications [34]. Here we detected the effect of SMG on DNA damage using the alkaline comet assay. As shown in Fig 1C, there were significantly increased DNA lesions in *Rad9* ^*-/-*^ MES cells cultured under SMG for 1 day over those maintained under 1G for 1 day, and this difference disappeared after 5 days of culture under SMG. There was not a significant difference in DNA lesions between 1G-treated and SMG-treated *Rad9* ^*+/+*^ MES cells for either 1 or 5 days of culture. This pattern was similar to those of both neutral comet assay and γ-H2AX staining mentioned above. Furthermore, in *Rad9* ^*-/-*^ MES cells cultured under SMG for 1 day, DSB levels measured by neutral comet assay increased 1.8 times (2.79/1.52) while DNA lesions measured by alkaline comet assay increased 2.7 times (3.86/1.44) relative to those of cells cultured under 1G for 1 day. These results suggest that SMG also induces single strand breaks and/or base modifications besides DSBs in *Rad9* ^*-/-*^ MES cells at the early stage of SMG treatment.

The ectopic expression of mouse Rad9 in *Rad9* ^*-/-*^ MES cells at a level comparable to that in *Rad9* ^*+/+*^ MES cells rescued the sensitivity of *Rad9* ^*-/-*^ MES cells to DNA-damaging agents to the level of *Rad9* ^*+/+*^ MES cells [33]. The role of Rad9 in preventing SMG-induced DNA lesions was confirmed by the experiment in which the induction of DSBs under SMG was suppressed by ectopically expressing mRad9 in *Rad9* ^*-/-*^ MES cells (Fig 1D). There was not a significant difference in DNA lesions between the 1G group and the SMG group.

One of the endpoint responses to DSBs is apoptosis. Here we examined whether SMG could induce apoptosis in MES cells, especially in *Rad9* ^*-/-*^ MES cells which contained significantly more DNA lesions for the first three days under SMG. We found that the percentage of apoptotic *Rad9* ^*-/-*^ MES cells was significantly higher in cells under SMG for 1 day than that in cells under 1G for 1 day (2.43% versus 1.64%, P = 0.03), while this phenomenon did not occur for *Rad9* ^*+/+*^ MES cells (Fig 1E). The result indicates that *Rad9* ^*-/-*^ MES cells are more susceptible of SMG-induced apoptosis than *Rad9* ^*+/+*^ MES cells.

**Fig 2:**
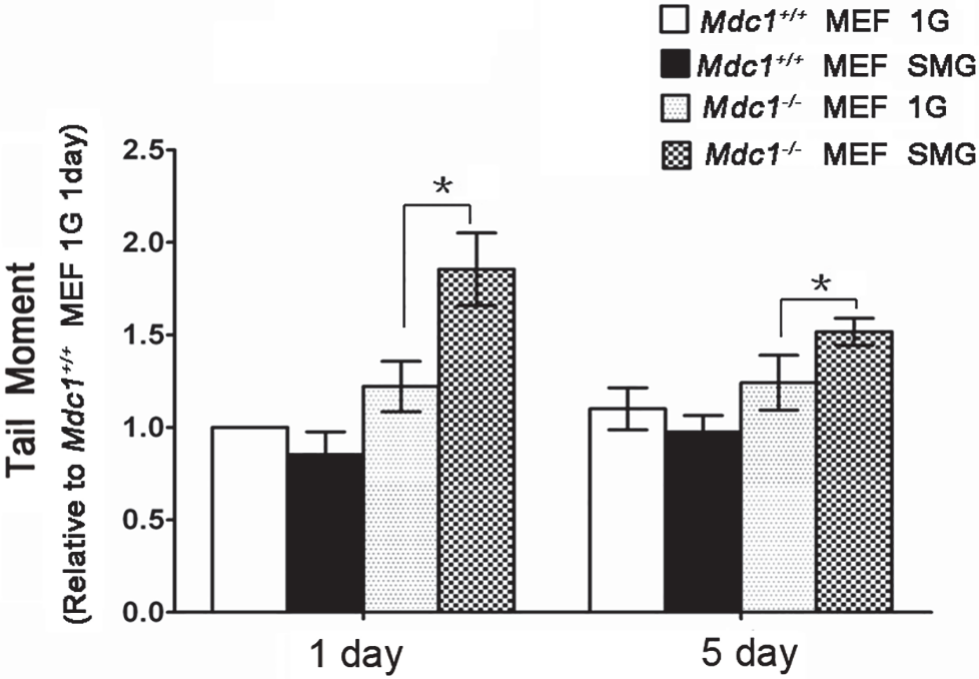
Effects of SMG on DNA damage in *Mdc1* ^*+/+*^ and *Mdc1* ^*-/-*^ MEF cells. Evaluation of DNA double strand break by neutral comet assay in *Mdc1* ^***+/+***^ and *Mdc1* ^***-/-***^ MEF cells cultured under 1G or SMG condition for 1 or 5 days. At least 50 cells for each condition were scored for comet tail moment. The data represent mean ± SD of three independent experiments. Student’s t test, *P<0.05.

We wondered if these phenomena could also occur to the deletion of other DNA repair genes and/or other types of cells such as *Mdc1* deletion in MEF. Mdc1 plays a critical role in the response to DNA DSBs [13, 14] and *Mdc1*-deficient mice have been shown to be hypersensitive to IR [29]. As shown in Fig 2, *Mdc1* ^*-/-*^ MEF cells cultured under SMG for 1 day contained significantly more DSBs than those under 1G. Similar to *Rad9* ^*-/-*^ MES cells, the SMG-induced DSBs in *Mdc1* ^*-/-*^ MEF cells attenuated to a significantly lower level after 4 additional days of culture under SMG, although the reduced level of DSBs was still statistically higher than that under 1G condition. There was not a significant difference in DSB levels between the 1G group and the SMG group in *Mdc1* ^*+/+*^ MEF cells for either 1 day’s or 5 days’ culture.

### Roles of Rad9 and Mdc1 to inhibit ROS production in DDR deficient cells experiencing SMG

**Fig 3:**
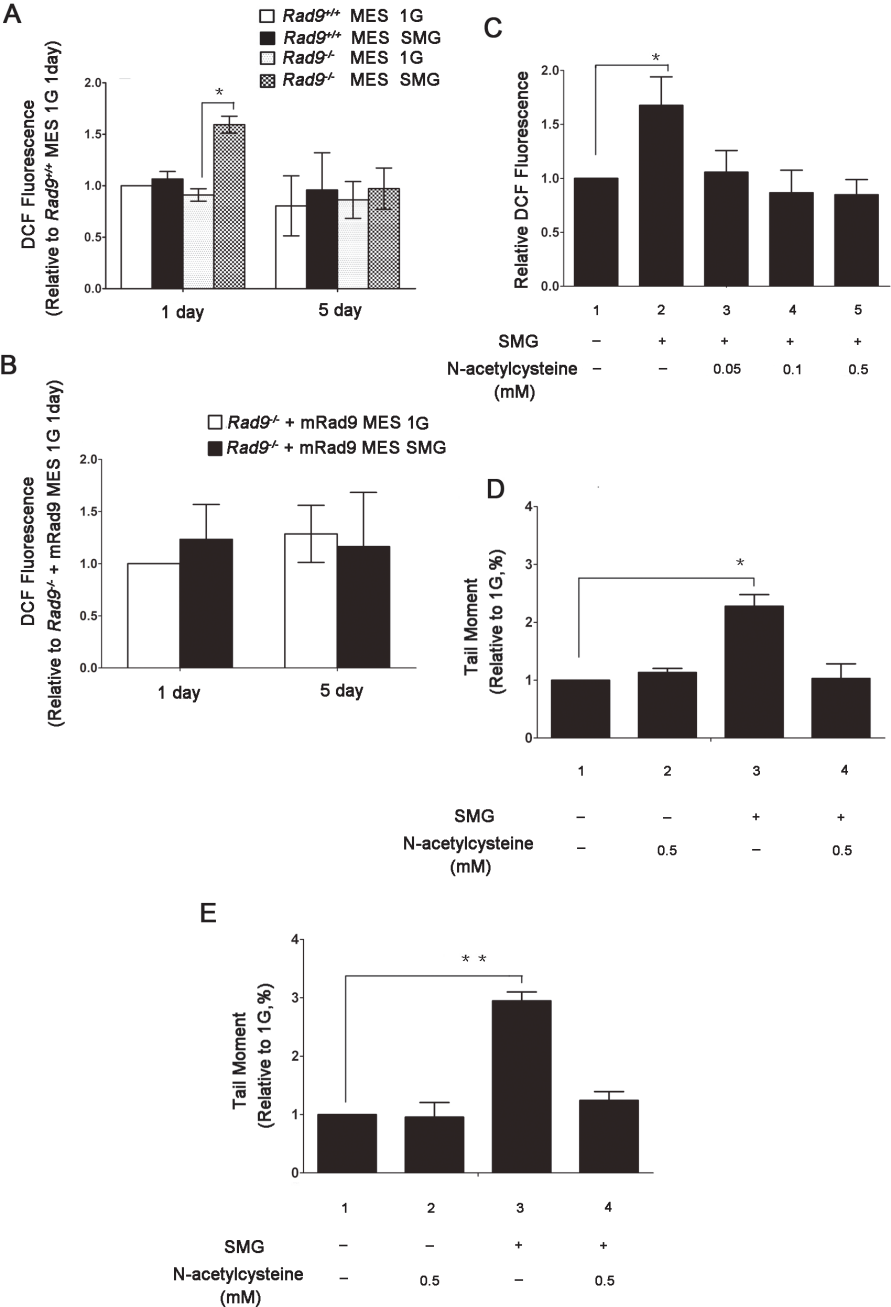
Effects of SMG on the generation of endogenous reactive oxygen species(ROS)in *Rad9* ^*+/+*^ and *Rad9* ^*-/-*^ MES cells. (A) Flow cytometric analysis of ROS activity in *Rad9* ^***+/+***^ and *Rad9* ^***-/-***^ MES cells exposed to 1G or SMG condition for 1 or 5 days. (B) Flow cytometric analysis of ROS activity in *Rad9* ^***-/-***^ *+Rad9* MES cells exposed to 1G or SMG condition for 1 or 5 days. (C) N-acetylcysteine inhibited SMG-induced increase of ROS formation in *Rad9* ^***-/-***^ MES cells. *Rad9* ^***-/-***^ MES cells were mock-treated or treated with 0.05, 0.1 or 0.5 mM N-acetylcysteine under SMG for 1 day. (D) Evaluation of DNA damage by alkaline comet assay in *Rad9* ^***-/-***^ MES cells mock-treated or treated with 0.5 mM N-acetylcysteine under 1G of SMG condition for 1 day. (E) Evaluation of DNA damage by neutral comet assay in *Rad9* ^***-/-***^ MES cells mock-treated or treated with 0.5 mM N-acetylcysteine under 1G of SMG condition for 1 day. The data represent mean ± SD of three independent experiments. Student’s t test *P<0.05, **P<0.01.

ROS can inflict DNA lesions [37], and SMG can generate ROS in some cell types such as neuronal origin rat PC12 cells [38]. In the last section we showed that SMG induced DNA lesions in *Rad9* ^*-/-*^ MES cells and *Mdc1* ^*-/-*^ MEF cells. We wondered if SMG induced ROS, which then caused DNA lesions in *Rad9* ^*-/-*^ MES cells and *Mdc1* ^*-/-*^ MEF cells. We monitored total intracellular ROS using Dichloro-dihydro-fluorescein diacetate (DCFH-DA) fluorescent probe; the fluorescence intensity is proportional to the ROS level within a cell (Refer to Materials and Methods). As shown in Fig 3A, the ROS level was significantly higher in *Rad9* ^*-/-*^ MES cells cultured under SMG for 1 day than that in cells under 1G, and the SMG-enhanced ROS in cells cultured for 5 days was reduced to a level which was not statistically different from that in cells under 1G. For *Rad9* ^*+/+*^ MES cells, the ROS level in cells cultured under SMG for either 1 or 5 days was slightly higher, but not statistically significantly different, from that in cells under 1G. Furthermore, for *Rad9* ^*-/-*^+mRad9 MES cells, there was not a significant difference in ROS production between the cells cultured under SMG and under 1G for either 1 or 5 days (Fig 3B). Thus, the pattern of the intracellular ROS concentration was closely correlated with that of SMG-induced DNA lesions, indicating that Rad9 is required for preventing ROS production MES cells experiencing SMG.

To check whether the SMG-induced ROS is responsible for the SMG-induced DNA lesions in *Rad9* ^*-/-*^ MES cells we used the ROS scavenger N-acetylcysteine to remove ROS and then measured DNA DSBs in *Rad9* ^*-/-*^ MES cells. N-acetylcysteine at 0.05, 0.1 or 0.5 mM concentrations was able to effectively suppress SMG-induced ROS production (Fig 3C). We used 0.5 mM N-acetylcysteine to reduce ROS and tested the effect of reduced ROS on SMG-induced DNA lesions. Without 0.5 mM N-acetylcysteine in culture, the DSB levels in *Rad9* ^*-/-*^ MES cells cultured under SMG for 1 day were significantly higher than those in cells maintained under 1G for 1 day (Fig 3D, between lanes 1 and 3, p < 0.05), consistent with what we observed above (Fig 1A and 1C). In the presence of 0.5 mM N-acetylcysteine in culture, there was not a significant difference in DSB levels between *Rad9* ^*-/-*^ MES cells cultured under 1G and those cultured under SMG (Fig 3D, between lanes 1 and 4, p > 0.05). Similar results were also obtained using alkaline comet assay to detect DNA lesions (Fig 3E). These data suggest that SMG inflicted DSBs as well as other DNA lesions by inducing ROS in *Rad9* ^*-/-*^ MES cells.

**Fig 4:**
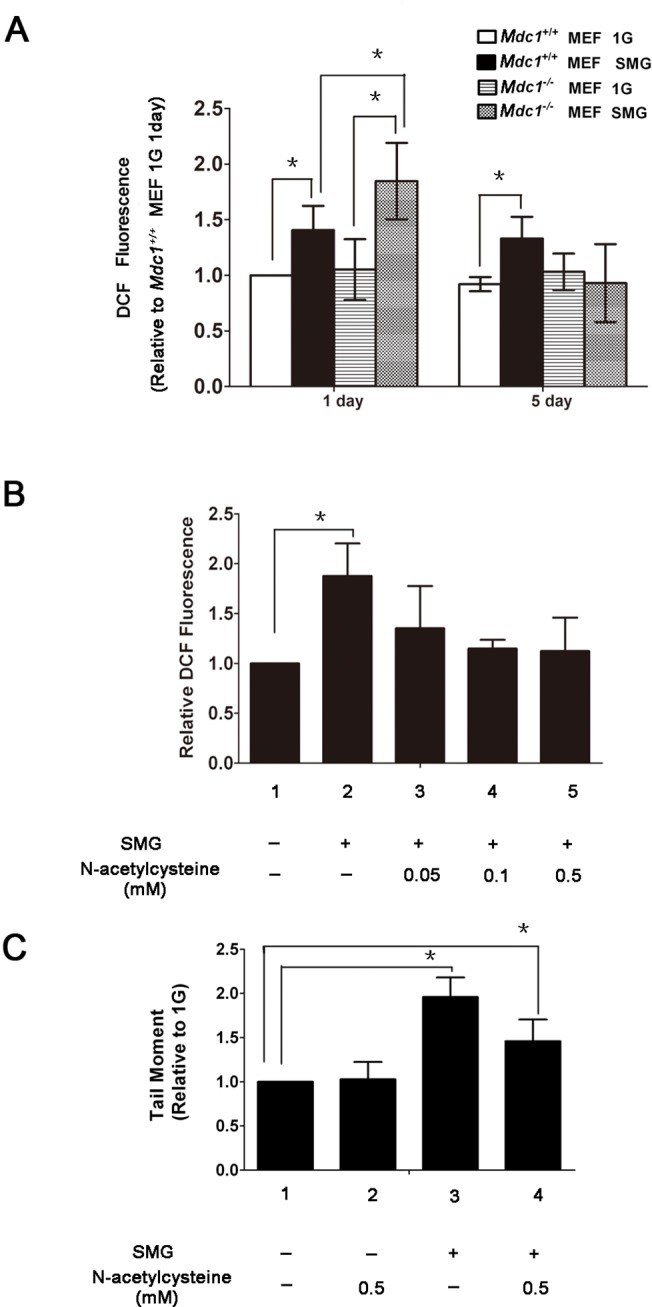
Effects of simulated microgravity on the generation of endogenous reactive oxygen species (ROS) in *Mdc1* ^*+/+*^ and *Mdc1* ^*-/-*^ MEF cells. (A) Flow cytometric analysis of ROS activity in *Mdc1* ^***+/+***^ and *Mdc1* ^***-/-***^ MEF cells exposed to 1G or SMG condition for 1 or 5days. (B) N-acetylcysteine inhibited SMG-induced ROS formation in *Mdc1* ^***-/-***^ MEF cells. *Mdc1* ^***-/-***^ MEF cells were mock-treated or treated with 0.05, 0.1 or 0.5 mM N-acetylcysteine under SMG for 1 day. (C) Evaluation of DNA damage by neutral comet assay in *Mdc1* ^***-/-***^ MEF cells mock-treated or treated with 0.5 mM N-acetylcysteine under 1G of SMG condition for 1 day. The data represent mean ± SD of three independent experiments. Student’s t test, *P<0.05.

We also observed that the ROS level was significantly higher in *Mdc1* ^*-/-*^ MEF cells cultured under SMG for 1 day than that in cells under 1G for 1 day, and SMG-induced ROS in *Mdc1* ^*-/-*^ MEF cells cultured for 5 days reduced to a level which was not statistically different from that in cells under 1G, a phenomenon obviously different from that of *Mdc1* ^*+/+*^ MEF cells (Fig 4A). Another difference from MES cells is that a significantly higher ROS level in cells under SMG than that in cells under 1G after 1 or 5 days of culture was observed in *Mdc1* ^*+/+*^ MEF cells (Fig 4A), although SMG did not induce DSBs in these cells (Fig 2). It is noteworthy that the SMG-induced ROS level in *Mdc1* ^*-/-*^ MEF cells was significantly higher than that in *Mdc1* ^*+/+*^ MEF cells after one day of culture, suggesting that Mdc1 counteracts the ROS production induced by SMG, but does not completely prevent the ROS production which is different from the role of Rad9 in *Rad9* ^*-/-*^ MES cells.

We also used the ROS scavenger N-acetylcysteine to reduce ROS levels and then measured DNA DSBs in *Mdc1* ^*-/-*^ MEF cells. N-acetylcysteine at 0.05, 0.1 and 0.5 mM concentrations was able to effectively suppress SMG-induced DSBs (Fig 4B). We chose the concentration of 0.5 mM N-acetylcysteine to remove ROS and tested the effect of ROS removal on SMG-induced DNA lesions. Without 0.5 mM N-acetylcysteine in culture, the DSB level in *Mdc1* ^*-/-*^ MEF cells under SMG for 1 day was significantly higher than that under 1G for 1 day (Fig 4C, lane 1 versus lane 3, p < 0.05), which was consistent with what we observed above (Fig 2). The DSB level in the group cultured under SMG combined with 0.5 mM N-acetylcysteine treatment was significantly lower than that in the group cultured under SMG alone (Fig 4C, lane 3 versus lane 4, p < 0.05), although still significantly higher than that in the group cultured under 1G (Fig 4C, lane 1 versus lane 4, p < 0.05). That is, 0.5 mM N-acetylcysteine could effectively suppress SMG-induced ROS generation in *Mdc1* ^-/-^ MEF cells, but it only partly decreased SMG-induced DNA damage.

### Effect of SMG on ROS generation enzymes and scavengers

**Fig 5:**
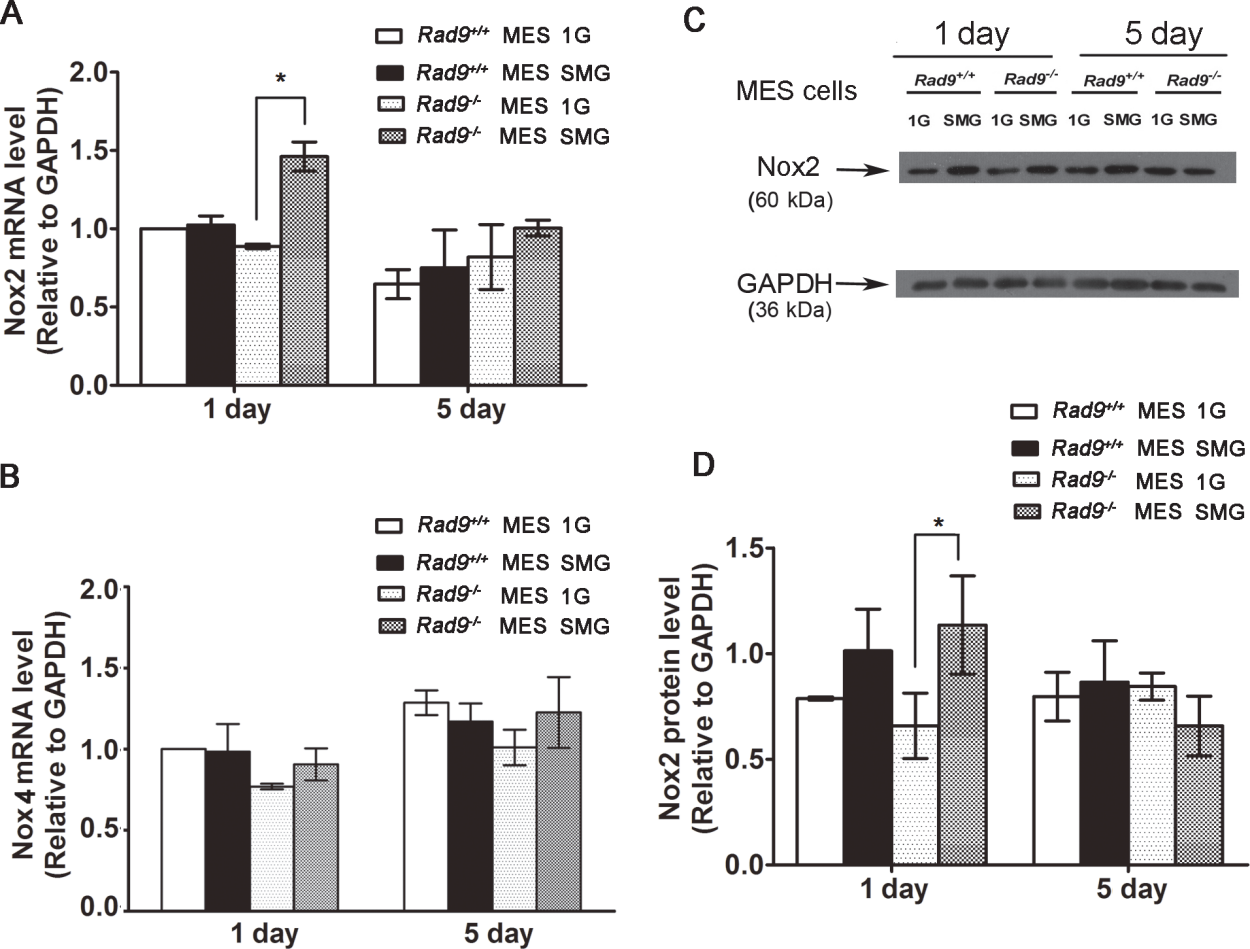
Effects of SMG on NADPH oxidase2 (Nox2) and NADPH oxidase4 (Nox4) expression in *Rad9* ^*+/+*^ and *Rad9* ^*-/-*^ MES cells. (A) Quantitative real-time PCR analysis of Nox2 mRNA expression in *Rad9* ^***+/+***^ and *Rad9* ^***-/-***^ MES cells exposed to 1G or SMG condition for 1 or 5 days. The expression levels of Nox2 were normalized to the endogenous control GAPDH expression. (B) Quantitative real-time PCR analysis of Nox4 mRNA expression in *Rad9* ^***+/+***^ and *Rad9* ^***-/-***^ MES cells exposed to 1G or SMG condition for 1 or 5 days. The expression levels of Nox4 were normalized to the endogenous control GAPDH expression. (C) Western blot analysis of Nox2 protein expression in *Rad9* ^***+/+***^ and *Rad9* ^***-/-***^ MES cells exposed to 1G or SMG condition for 1 or 5 days. GAPDH was used as an internal control. The representative results of three independent experiments were shown. (D) Quantitative comparison of Nox2 expression. Data were derived from three independent experiments. The expression levels of Nox2 protein were normalized to the endogenous control GAPDH protein expression. The data represent mean ± SD of three independent experiments. Student’s t test, *P<0.05.

**Fig 6:**
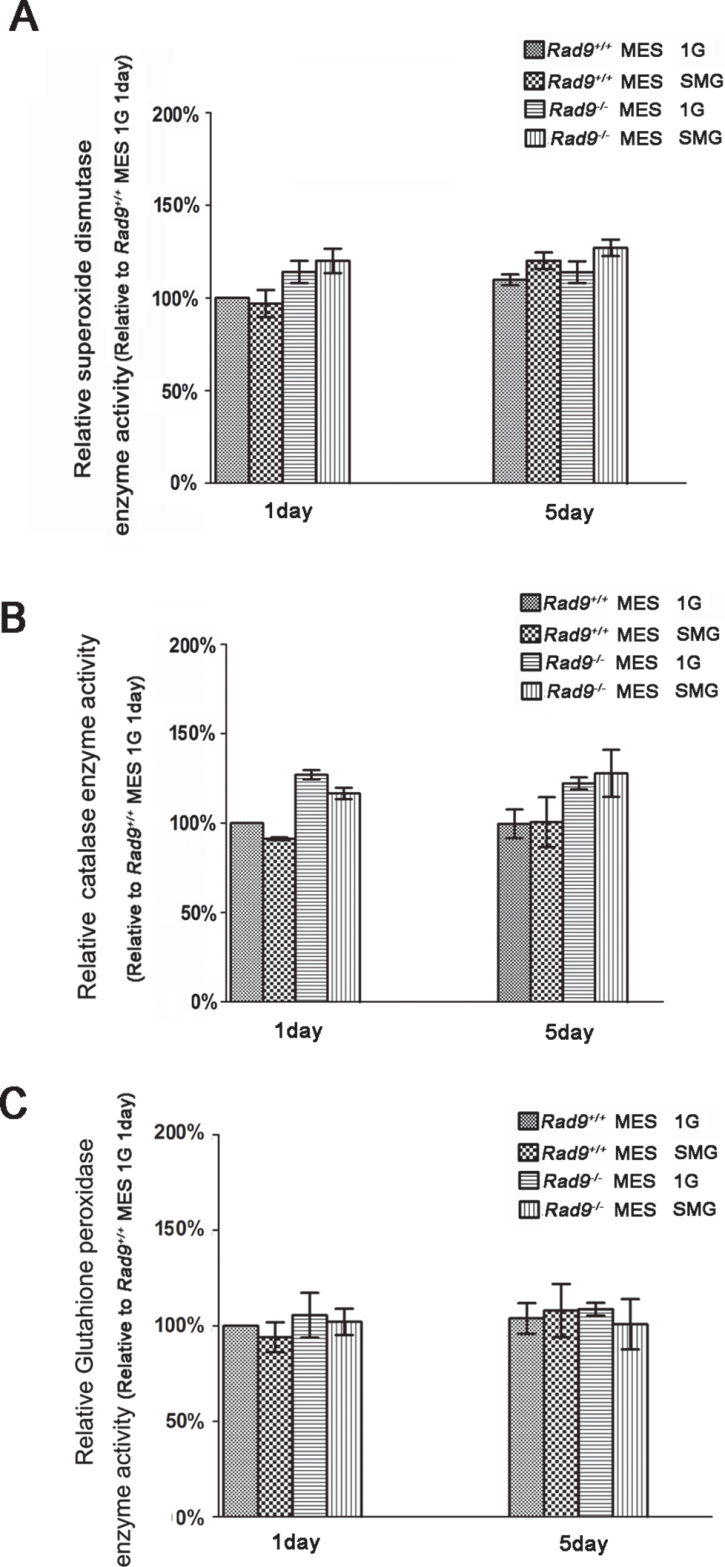
Effects of SMG on antioxidant enzyme activity in MES cells. *Rad9* ^***+/+***^ MES cells and *Rad9* ^***-/-***^ MES cells were cultured under 1G and SMG for 1 and 5 days, respectively. The activities of the antioxidant enzymes in MES cell lysates were determined. (A) Histograms of superoxide dismutase enzyme activity. (B) Histograms of catalase enzyme activity. (C) Histogram of Glutahione peroxidase. The data represent mean ± SD of three independent experiments.

SMG-induced ROS can be derived from increasing the activities of ROS-generating enzymes or the activities of ROS scavengers [38]. NADPH oxidase (Nox) enzymes are a main source of cellular ROS production. There are seven Nox enzymes [39]. Nox2, also known as gp91phox, is the prototype NADPH oxidase and appears as the most widely distributed Nox isoform. Nox1, Nox2 and Nox4 were found to express at low levels in MES cells [40]. In this study, mRNA levels of Nox1, Nox2 and Nox4 in MES cells were analyzed by qRT-PCR. Nox2 mRNA was significantly induced in Rad9^-/-^ MES cells after 1 day of culture under SMG, and after 5 days of culture under SMG was reduced to the levels in the same cells cultured 1 or 5 days under 1G (Fig 5A). The Nox2 mRNA levels in *Rad9* ^*+/+*^ MES cells remained almost the same between 1G and SMG conditions either cultured for 1 day or 5 days, although the levels of Nox2 mRNA were slightly lower in the cells after 5 days’ culture than those after 1 day culture. There were not significant differences in Nox4 mRNA levels in MES cells cultured under 1G and SMG (Fig 5B). We were unable to detect Nox1 mRNA. To check if the enhanced Nox2 mRNA can be translated into Nox2 protein, we analyzed Nox2 by Western blotting. As shown in Fig 5C and 5D, Nox2 expression was significantly higher in Rad9^-/-^ MES cells cultured under SMG for 1 day than that under 1G. Enhanced Nox2 expression in these cells cultured for 5 days under SMG was reduced to a level that was not statistically different from that in cells under 1G. As for *Rad9* ^*+/+*^ MES cells, there was no significant difference in Nox2 expression between the SMG group and the 1G group for either 1 or 5 days of treatment. Antioxidant activity is a self-protective cellular mechanism. The tolerance to ROS may correlate with the state of intracellular antioxidant enzymes such as superoxide dismutase (SOD), glutathione peroxidase (GSH-Px), and catalase (CAT) [41]. SMG did not affect the activities of ROS-scavenging enzymes, including SOD (Fig 6A), CAT (Fig 6B) and GSH-Px (Fig 6C) in both *Rad9* ^*+/+*^ and *Rad9* ^*-/-*^ MES cells for 1 or 5 days of treatment. The above data suggest that SMG-induced Nox2 expression makes a contribution to SMG-induced ROS production in Rad9^-/-^ MES cells.

**Fig 7:**
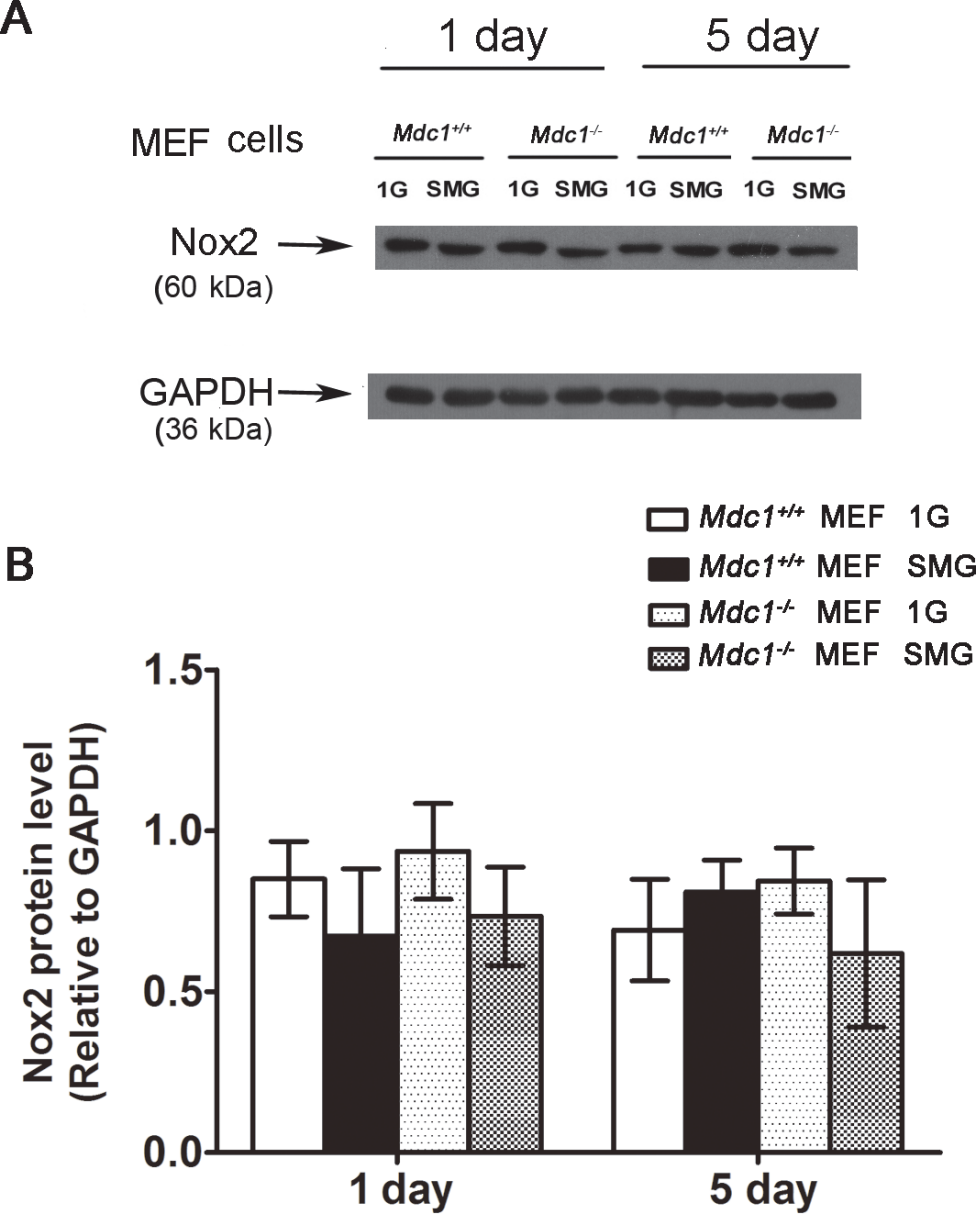
Effects of SMG on NADPH oxidase2 (Nox2) expression in *Mdc1* ^*+/+*^ and *Mdc1* ^*-/-*^ MEF cells. (A) Western blot analysis of Nox2 protein expression in *Mdc1* ^***+/+***^ and *Mdc1* ^***-/-***^ MEF cells exposed to 1G or SMG condition for 1 or 5days. GAPDH was used as an internal control. The representative results of three independent experiments were shown. (B) Quantitative comparison of Nox2 expression. Data were derived from three independent experiments as in A). The expression levels of Nox2 were normalized to the endogenous control GAPDH expression. The data represent mean ± SD of three independent experiments. Student’s t test, *P<0.05.

We also detected Nox2 expression in MEF cells using Western blotting analysis. Although SMG-induced ROS production could be observed in *Mdc1* ^*+/+*^ MEF cells after 1 or 5 days of treatment, as well as in *Mdc1* ^-/-^ MEF cells after 1 day of treatment, SMG did not have effect on Nox2 expression in either *Mdc1* ^*+/+*^ or *Mdc1* ^-/-^ MEF cells after 1 or 5 days of treatment (Fig 7A and 7B). In summary, ROS is only partially responsible for SMG-induced DNA damage in *Mdc1* ^-/-^ MEF cells, and SMG-induced ROS production in both *Mdc1* ^*+/+*^ and *Mdc1* ^-/-^ MEF cells does not correlate with its effects on Nox2 expression. These findings are different from what we observed in *Rad9* ^*+/+*^ and *Rad9* ^*-/-*^ MES cells.

### Roles of Rad9 and Mdc1 in reducing cell number expansion during SMG treatment

**Fig 8:**
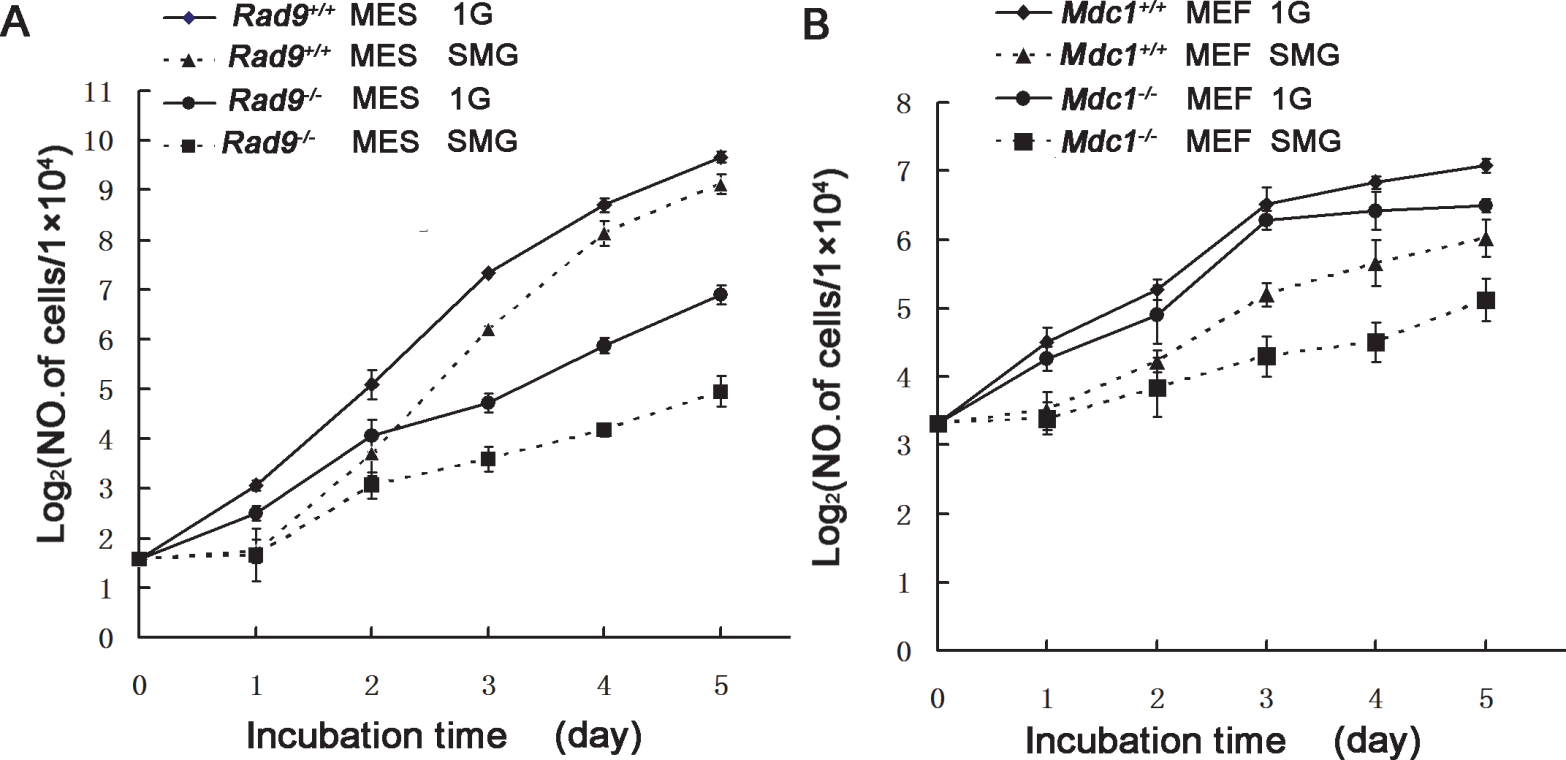
Cell Number Expansion analysis of MES and MEF cells after five days incubation under 1G or SMG. (A) The initial MES cells seeding number was 3×10^4^. The cell doubling curve was generated by dividing the cell number by10^4^ and then transforming the values to logarithm base2. (B) The initial MEF cells seeding number was 10^5^. The cell doubling curve was generated by dividing the cell number by10^4^ and then transforming the values to logarithm base2. The data represent mean ±SD of three independent experiments.

As mentioned above, we observed effects of SMG on mammalian cell DNA damage in *Rad9* ^-/-^ MES and *Mdc1* ^-/-^ MEF cells but not in the corresponding wild type cells. We also saw effect of SMG in inducing apoptosis in *Rad9* ^-/-^ MES but not in *Rad9* ^+/+^ MES. These effects could affect the total cell number in culture. Here we described the effect using the rate of cell number expansion which is the sum of cell proliferation and death through various ways including apoptosis. As for *Rad9* ^*+/+*^ and *Rad9* ^*-/-*^ MES cells cultured under 1G, the number of *Rad9* ^*-/-*^ MES cells was significantly lower than that of *Rad9* ^*+/+*^ MES cells after 1 to 5 days under SMG, consistent with previous reports [33, 42, 43]. As shown in Fig 8A, the slope of the cell doubling curve for both *Rad9* ^*+/+*^ and *Rad9* ^*-/-*^ MES cells under SMG was lower than that for the cells under 1G for 1 day, but was almost identical to the slopes for cells cultured under 1G from 2 to 5 days, suggesting that the effect of simulated SMG on cell expansion was acutely sensed in the early stage of treatment and counterbalanced afterwards in both *Rad9* ^*+/+*^ and *Rad9* ^-/-^MES cells, thus that Rad9 does not play a role in the effect of SMG on cell number expansion.

*Mdc1* ^*+/+*^ MEF cells grew faster compared to *Mdc1* ^-/-^ MEF cells under 1G. The expansion of both genotype cells was sensitive to SMG. However, the cell number expansion of *Mdc1* ^-/-^ MEF cells was affected much more than that of *Mdc1* ^*+/+*^ MEF cells by SMG (Fig 8B). The rate of cell number expansion of *Mdc1* ^*+/+*^ MEF cells under SMG for the first day was significantly reduced, but later was close to that of cells under 1G, indicating that the acute effect of SMG is sensed by the cells mainly during the first day culture. The same effect of SMG also happened to *Mdc1* ^-/-^ MEF cells. However, the dynamics of the expansion rate of the cells under SMG was different from that of *Mdc1* ^*+/+*^ MEF cells. The expansion rate of *Mdc1* ^-/-^ MEF cells was still affected by SMG from day 1 to day 3, although not as much as for the first day. In contrast to the first three days, the expansion of *Mdc1* ^-/-^ MEF cells from day 3 to day 5 was greater under SMG than 1G, and this was not because *Mdc1* ^-/-^ MEF cells grew faster but because *Mdc1* ^*+/+*^ MEF cells grew slower during the last two days. The slower growth of *Mdc1* ^*+/+*^ MEF cells was most likely due to the crowdedness and contact inhibition during the last two days in culture (data not shown).

In summary, SMG slowed down the cell number expansion acutely during the first day of SMG exposure in both MES and MEF cells, either with intact *Rad9/Mdc1*, or their deficient versions. After the first day, Rad9 does not play a role in the slowdown of MES cell number expansion. However, Mdc1 still plays a small but tangible role in the slowdown of MEF cell number expansion…

## Discussion

In this study, we investigated the effect of SMG on DNA damage using *Rad9* ^-/-^ MES and *Mdc1* ^-/-^ MEF cells, as well as the corresponding wild type cells. We observed that SMG induced DNA damage in *Rad9* ^-/-^ MES and *Mdc1* ^-/-^ MEF cells but not in the corresponding wild type cells (Figs 1 and 2). As the culture time under SMG was lengthened, the increase in DNA lesions in *Rad9* ^-/-^ MES cells gradually decreased, and disappeared after 5 days of culture under SMG (Fig 1). A similar pattern of change in DNA DSBs also happened in *Mdc1* ^-/-^ MEF cells (Fig 2). The enhancement of DNA damage was mediated through SMG-induced ROS generation in *Rad9* ^-/-^ MES cells (Fig 3) and partially in *Mdc1* ^-/-^ MEF cells (Fig 4). Increased Nox2 transcription may contribute greatly to SMG-induced ROS generation in *Rad9* ^-/-^ MES cells (Fig 5).

Previously, we reported that SMG alone was unable to induce DNA DSBs in wild type MES cells after 2 days of exposure [5]. In this study, we systematically investigated the effects of SMG on DNA DSBs in *Rad9* ^*+/+*^ MES cells, and found that SMG alone did not induce DNA DSBs in wild type MES cells after either short (1 day) or long (5 days) durations of exposure to SMG. Although there were slight increases in DSBs in wild type MES cells cultured for 3 or 4 days under SMG, the SMG-induced DSBs did not reach levels that were statistically significantly different from those in cells cultured for the same time periods under 1G. We did not observe SMG-induced single strand or base modifications in wild type MES cells cultured for either 1 or 5 days of SMG exposure (Fig 1C). We did not see enhanced DSBs in wild type MEF cells either after 1 or 5 days of SMG exposure (Fig 2). These results obviously contrast with the observation of SMG-induced DNA lesions in lymphocytes and human retinal pigment epithelial cells under SMG [6–8]. Therefore, whether DNA lesions are induced by SMG depends on the cell type.

One of the explanations for the phenomena that the induction of DNA lesions by SMG depends on the cell type is that SMG is a weak genotoxin and its effect on DNA damage can be revealed only when the ability of DNA repair of a cell type is not strong enough to counter the level of DNA lesions in the cell. Accordingly, DNA repair capacities and the intracellular concentrations of ROS that can cause DNA lesions differ in different types of cells [44]. A strategy to create cells with a weaker capacity for DNA repair is to delete genes with functions in DDR. Both Rad9 and Mdc1 plays important roles in DDR, and in this study we tested whether SMG could induce DNA lesions in *Rad9* ^-/-^ MES and *Mdc1* ^-/-^ MEF cells, but not in their wild type counterparts. The positive results of these tests suggest that SMG is a weak genotoxic stress.

We reasoned that SMG could induce DNA lesions either by raising the level of factors that cause DNA lesions or by impairing the DDR system. All living cells generate ROS, so we examined the levels of ROS in cells cultured under SMG and 1G. We observed time-dependent SMG-induced ROS generation in *Rad9* ^-/-^ MES cells but not in wild type MES cells. There was no significant difference in ROS production between *Rad9* ^*-/-*^ and wild type MES cells cultured under 1G for 1 day. Therefore, both Rad9 deletion and SMG were required for the enhancement of ROS, and ROS was reduced to a level close to that of cells under 1G when *Rad9* ^-/-^ MES cells were cultured under SMG for 5 days (Fig 3A). One explanation is that SMG can raise ROS levels and Rad9 may help reduce ROS concentrations. In the absence of Rad9, when an enhanced level of ROS is sensed, another mechanism is activated and suppresses ROS to a level close to that of cells under 1G. Our data demonstrated that Nox2 transcription also changed in a time-dependent manner similar to the changes in ROS and DSBs (Fig 5A–5C), suggesting that Rad9 plays a role in suppressing Nox2 transcription. Interestingly, Yin et al. proposed that modulation of gene transcription is a mechanism by which Rad9 controls multiple cellular processes[45]. How Rad9 regulates Nox2 expression deserves further investigation. The similar pattern in the levels of DSBs and ROS in *Rad9* ^-/-^ MES cells also suggests that the increased concentration of ROS is responsible for the increased DSBs, and this supposition was supported by the observation that the DSBs in *Rad9* ^-/-^ MES cells cultured under SMG for 1 day were suppressed by the ROS scavenger N-acetylcysteine to a level nearly identical to that of the same cells under 1G.

We tried to answer the question of whether ROS levels could also be raised in cells other than MES cells and in a genetic background deficient for another DDR gene. We found that 1 day culture under SMG induced a significantly higher level of ROS in *Mdc1* ^*-/-*^ MEF cells, but also in wild type MEF cells although the increased level of ROS in the wild type cells was significantly lower than that in *Mdc1* ^*-/-*^ MEF cells (Fig 4A). These data suggest that Mdc1 is partially responsible for inhibiting the enhancement of intracellular ROS concentration induced by SMG and that the SMG-induced ROS in wild type MEF cells, in contrast to MES cells, cannot be suppressed completely by an intrinsic mechanism. After 5 days of culture, the ROS level in *Mdc1* ^*-/-*^ MEF cells under SMG returned to that of cells under 1G, while the ROS level in wild type MEF cells under SMG was still significantly higher than that of cells under 1G. Similar to the pattern for *Rad9* ^-/-^ MES cells, the ROS level increased in *Mdc1* ^*-/-*^ MEF cells under SMG for 1 day and then returned to that of cells under 1G; however, the dynamic change of the ROS concentration in wild type MEF cells was different from MES wild type cells. We did not observe that SMG had an effect on Nox2 transcription in either *Mdc1* ^*+/+*^ or *Mdc1* ^-/-^ MEF cells cultured for 1 or 5 days. Thus the mechanisms of SMG-induced ROS production between MEF cells and MES cells are different, and Mdc1 is only partially responsible for the suppression of the SMG-induced ROS in MEF cells. It will be worthwhile to check if other DDR genes are also involved in this process and what is the outcome in other types of cells.

Another important question is whether the ROS induced by SMG in MEF cells is responsible for the SMG-induced DSBs. As described above, the SMG-induced ROS is responsible for the majority, if not all, of the SMG-induced DSBs in *Rad9* ^-/-^ MES cells, but the SMG-induced ROS is only partially responsible for the DSBs caused by SMG in *Mdc1* ^-/-^ MEF cells as the ROS scavenger N-acetylcysteine suppressed the ROS level in cells cultured for 1 day under SMG to that of cells under 1G but was unable to reduce the DSB level to that of cells under 1G. This proposition is also supported by the observation that culturing *Mdc1* ^-/-^ MEF cells under SMG for 5 days returned the ROS level to that of cells under 1G but could not reduce the SMG-induced DSBs to that of cells under 1G. These data suggest that there are other mechanism(s) through which SMG affects the level of DSBs in *Mdc1* ^-/-^ MEF cells. These mechanisms could involve repair, cell cycle control and/or signal transduction, three important elements in the DDR. We are unable to answer why MEF cells behaved differently from MES cells, or why Mdc1 deficient cells behaved differently than Rad9 deficient cells in response to SMG treatment. It would be ideal if *Rad9* ^-/-^ MEF cells and *Mdc1* ^-/-^ MES cells were available to compare Rad9 and Mdc1 in the same cell type, and to compare MEF and MES cells deficient for Rad9 or Mdc1.

It should be noticed that although SMG induced significantly higher levels of ROS in both *Mdc1* ^*+/+*^ and *Mdc1* ^*-/-*^ MEF cells, we only observed increased DSB levels in *Mdc1* ^*-/-*^ MEF cells but not in *Mdc1* ^*+/+*^ MEF cells. This indicates that wild type MEF cells whose DDR system is intact can effectively maintain genomic stability in response to the enhanced level of ROS induced by SMG. The different responses of MES and MEF cells to SMG indicates the importance of including multiple cell types in this type of research.

We observed the adaptations of both MES and MEF cells to SMG in several aspects. SMG induced acute effects on cells cultured for the first day, then the effects were gradually reduced afterwards. We name the reduction of these effects after culturing these cells for more than one day as adaptation. As for cell number expansion, the adaptation occurred in both MES and MEF cells, and in both wild type and *Rad9* or *Mdc1* deficient genetic backgrounds, and the adaptation was nearly complete after 5 days of culture as demonstrated by the nearly identical slopes of cell number expansion curves for cells under SMG and 1G (Fig 8). As for DNA lesions, the adaptation happened only to the cells with *Rad9* or *Mdc1* deletion (Figs 1 and 2). The adaptation of DNA lesions was complete for *Rad9* ^*-/-*^ MES cells, but not for *Mdc1* ^-/-^ MEF cells, within 5 days under SMG. As for ROS enhancement, a complete adaptation occurred in both *Rad9* ^*-/-*^ MES cells and *Mdc1* ^-/-^ MEF cells within 5 days under SMG, but not in wild type MEF cells (Fig 4A). Cell number expansion can be affected by the levels of DNA lesions and apoptosis. However, how ROS level affects cell number expansion is not understood. Whether adaptations of various cellular activities to microgravity actually happen in space warrants future investigations.
